# Highlighting Immune System and Stress in Major Depressive Disorder, Parkinson’s, and Alzheimer’s Diseases, with a Connection with Serotonin

**DOI:** 10.3390/ijms22168525

**Published:** 2021-08-07

**Authors:** Ana Salomé Correia, Armando Cardoso, Nuno Vale

**Affiliations:** 1 OncoPharma Research Group, Center for Health Technology and Services Research (CINTESIS), Rua Dr. Plácido da Costa, 4200-450 Porto, Portugal; anncorr07@gmail.com; 2 Institute of Biomedical Sciences Abel Salazar (ICBAS), University of Porto, Rua de Jorge Viterbo Ferreira 228, 4050-313 Porto, Portugal; 3 NeuroGen Research Group, Center for Health Technology and Services Research (CINTESIS), Rua Dr. Plácido da Costa, 4200-450 Porto, Portugal; cardosoa@med.up.pt; 4 Unit of Anatomy—Department of Biomedicine, Faculty of Medicine, University of Porto, Alameda Professor Hernâni Monteiro, 4200-319 Porto, Portugal; 5 Department of Community Medicine, Health Information and Decision (MEDCIDS), Faculty of Medicine, University of Porto, Al. Prof. Hernâni Monteiro, 4200-319 Porto, Portugal

**Keywords:** stress, immune system, major depressive disorder, Alzheimer’s disease, Parkinson’s disease, serotonin

## Abstract

There is recognition that both stress and immune responses are important factors in a variety of neurological disorders. Moreover, there is an important role of several neurotransmitters that connect these factors to several neurological diseases, with a special focus in this paper on serotonin. Accordingly, it is known that imbalances in stressors can promote a variety of neuropsychiatric or neurodegenerative pathologies. Here, we discuss some facts that link major depressive disorder, Alzheimer’s, and Parkinson’s to the stress and immune responses, as well as the connection between these responses and serotonergic signaling. These are important topics of investigation which may lead to new or better treatments, improving the life quality of patients that suffer from these conditions.

## 1. Introduction

**Figure 1:**
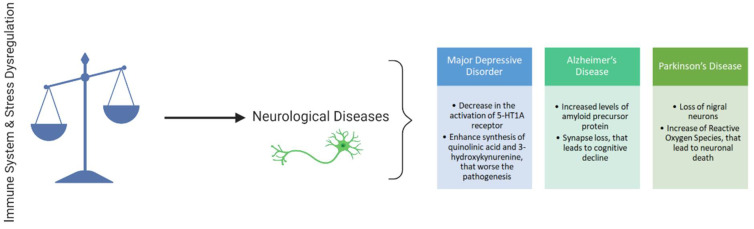
Examples of processes such as increased levels of the amyloid precursor protein, loss of nigral neurons, and decrease in the activation of 5-HT1A receptors are implicated as results of immune and stress dysregulation, worsening neurological diseases such as MDD, AD and PD. Created with BioRender.com.

There is growing recognition that both stress and immune responses are important factors in a variety of neurological disorders that are, in turn, interrelated with numerous variables, such as the number of neurotransmitters existing in the human body, at any given time [1,2] (Figure 1). Different responses to stress exist, depending on if it is acute or chronic. The first one is a physiological response, whereas the second one is, generally, harmful, dysregulating the typical and healthy stress response. Hereupon, uncontrollable, chronic stress may promote a variety of changes in the several components of the central nervous system, neuropsychiatric and neurodegenerative disorders [3,4]. Indeed, research in neuropsychiatry suggests that stress-related abnormalities have a relevant role in the pathogenesis of these kinds of diseases. Additional studies also state that imbalances in the stress response have a relevant role in neurodegenerative disorders [4].

**Figure 2:**
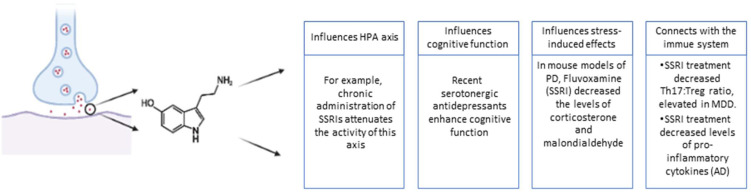
Serotonin, a neurotransmitter with key roles in the process of neuronal transmission, is also an important mediator in the stress and immune responses in several pathologies, such as MDD, AD and PD. In this figure, the chemical structure represents serotonin and the text summarizes some relevant functions of this neurotransmitter in the context of this article. Created with BioRender.com.

The serotonergic signaling in the brain is affected by several aspects of the stress response, and drugs that interfere with serotonergic pathways can influence the effects of stress [5]. An example that illustrates this relationship is an experiment carried out with cynomolgus monkeys which were separated by their resilience to stress as high, medium, and low resilience. In this study, animals more sensitive to stress showed less expression in several genes that are important for the normal functioning of the serotonergic system. These genes include *Plasmacytoma expressed transcript 1* (Pet-1), *tryptophan hydroxylase* (Tph2), *serotonin transporter* (SERT), and *serotonin 1A receptor* (5-HT1A). All these proteins are important for the development of brain serotonergic systems, production, transport, and serotonin functions, respectively. In turn, in this same study, there was an increase in the expression of corticotropin-releasing hormone (CRH) in animals more vulnerable to stress compared to those more resilient [6]. Hence, all these data suggest that serotonin and sensitivity to stress are involved. These effects of stress on the serotonergic system can be chronic and involve changes in gene and epigenetic expression, leading to changes in this system [7]. Additionally, stressors can enhance or suppress immune functions. A huge number of biological processes, such as stress, memory, sleep and feeding, are modulated by peripheral cytokines, a fact that highlights the existence of communication between the immune system and the brain [2]. Inflammatory changes in the brain are associated with dementia and depression, where serotonin also has some relevant roles (Figure 2). A well-known example is the depression associated with rheumatoid arthritis. Moreover, many of clinical studies have proved that depressed patients have raised blood levels of proinflammatory cytokines [8]. Furthermore, regarding Parkinson’s disease, there are also pieces of evidence supported by epidemiological, pharmacological, and genetic studies that inflammatory processes are crucial for the disease progression [9]. Thus, studying inflammatory processes, stress responses, and their connection to each other and neuronal processes, is crucial for a deeper understanding of the extremely relevant diseases, Major Depressive Disorder (MDD), Parkinson’s disease (PD), and Alzheimer’s disease (AD).

## 2. Stress Influence

### 2.1. In Major Depressive Disorder

A variety of body systems are involved in the stress response, of note the autonomic nervous system and hypothalamic-pituitary-adrenal (HPA) axis. The activation of the abovementioned axis results in the elevation of glucocorticoid levels [4,10]. CRH is involved in the HPA axis in a way that stimulates the hypophysis to release adrenocorticotropin hormone (ACTH) and, consequently, regulating glucocorticoids release and production. MDD is a serious mental illness, which has several hypotheses to explain its pathophysiology. However, low levels of serotonin in the central nervous system are one of the hypotheses most supported by the scientific community, despite the need for clarification [11]. This disease has a set of complex and sometimes unclear biological processes implicated in its etiology, including psychological stress; melancholia and atypical features are, respectively, linked to the hyperactivity of the stress system and its downregulation [2,12,13]. Evidence for a crucial interplay between the stress system and MDD appears in several aspects. It is known that antidepressants downregulate the HPA axis function and that by antagonizing CRH, there is a reduction of the stress response [2]. Highlighting the role of the stress system in MDD, about 50% of patients with depression show HPA axis dysfunction, resulting in impaired regulation of corticosteroid secretion. Additionally, elevated CRH mRNA expression in the hypothalamus is observed in patients with MDD, as well as increased levels in cerebrospinal fluid [4]. As mentioned above, by triggering the HPA axis, stress leads to the release of glucocorticoids. In a study in mice, this prolonged exposure to corticosterone (glucocorticoid in mice) resulted in morphological changes in neuronal dendrites, promoting atrophy [14,15]. In addition, synapse loss and neuronal death by glutamate excitotoxicity was observed [16,17,18]. All these findings correlate with cognitive decline, leading to pathologies such as MDD. There is also evidence that MDD relates to reduced volumes of hippocampus and prefrontal cortex, in which chronic stress plays an important role. Indeed, in studies with animal models of depression induced by stress, it was observed that within each episode of depression, a more pronounced reduction of the brain was observed [19]. Some experimental studies have also demonstrated that an elevation in stress in rats is correlated with a decrease in the functional way of the 5-HT1A receptor, activation of serotonin 2 receptor (5-HT2) and serotonin 1b receptor (5-HT1B). Thus, it is notorious that stress-induced changes affect the serotonergic system involved in depression [20]. Additionally, in stressed animals, it was observed that in vivo concentrations of brain metabolites decreased, particularly N-acetyl-aspartate (−13%), creatine and phosphocreatine (−15%), and choline-containing compounds (−13%). These effects of stress were prevented by the administration of tianeptine, an antidepressant drug [21]. In another study, MDD was associated with higher oxidative stress malondialdehyde levels and lower zinc and uric acid levels, in comparison to healthy controls [22]. Other studies point out that stress-associated epigenetic changes in the *human glucocorticoid receptor gene* (NR3C1) [23,24], *human serotonin transporter gene* (SLC6A4) [25,26], *brain-derived neurotrophic factor gene*, (BDNF) [27], *FK506 binding protein 5 gene* (FKBP5) [28], and *spindle and kinetochore associated complex 2 gene* (SKA2) [29], were found to be associated with a diagnosis of MDD. An outstanding question is whether stress dysregulation is a cause or a result of depression [4]. This is an interesting and promising focus of research.

Changes in the serotonergic system related to mood and anxiety disorders, as well as with reactivity to stress, have been widely discussed and are relevant topics. Several studies have focused on the relationship between selective serotonin reuptake inhibitors (SSRIs—drugs regularly used in the treatment of depression, which increase the extracellular concentration of serotonin) and various components of the HPA axis [5,30]. Several shreds of evidence show that the acute administration of fluoxetine (SSRI most clinically prescribed for MDD) leads to a very marked increase in the concentration of extracellular serotonin in the hypothalamus, including in the paraventricular nucleus which is enriched in CRH–containing neurons. Thus, evidence demonstrates that acute administration of this drug increases the activity of the HPA axis, including increased levels of CRH mRNA, increased expression of transcription factors, and increased concentration of ACTH and corticosterone in the plasma. However, these same studies have shown that serotonin can activate/inhibit the HPA axis in a dose-dependent manner, duration of action, and subtype of serotonergic receptor involved. For example, the administration of 5-HT1A agonists into the paraventricular nucleus inhibits the activity of the HPA axis at low dosages, causing the opposite effect at high dosages [31]. Besides, several pieces of evidence demonstrate that the chronic administration of SSRIs reduces the activity of the HPA axis, thus decreasing the plasma levels of ACTH, and of CRH mRNA in the paraventricular nucleus [32].

### 2.2. In Alzheimer’s Disease

Chronic stress is one risk factor associated with AD pathogenesis [33,34]. Several studies report that a variety of stressors raise the level of several pathologic features associated with AD, namely amyloid precursor protein, Aβ peptide, and intracellular neurofibrillary tangles. Additionally, loss of synaptic plasticity is also observed, as well as increases in hyperphosphorylated tau, intracellularly [33,35,36]. While the HPA axis is activated to a great extent in MDD, in AD, it is only moderately activated, which can be explained by the early damage in the hippocampus caused by this disease. Although cortisol and CRH can be involved in MDD, there is no conclusive evidence regarding if they are involved in the major irreversible damage that the hippocampus suffers during AD. That does not mean that cortisol does not play a role in AD as it has a strong interaction with the inflammatory response in AD [37]. The cortisol levels in plasma are increased with AD [37] and the levels of salivary cortisol are correlated with ongoing AD [38], adding to the fact that there is a linear relationship between levels of cortisol in plasma and cerebrospinal fluid (CSF). Additionally, neuronal loss was found in the hippocampus of rodents that are stressed/treated with corticosteroids [39]. It was expected that, during long periods of stress, the administration of glucocorticoids would damage the hippocampus, especially in the more elderly rodents. Under normal conditions, the HPA axis is inhibited by the hippocampus, so the damage that the hippocampus suffers will lead to an activation of this axis, further leading to more active production of glucocorticoid levels, accumulating damage in the hippocampus. The degree to which the HPA axis is activated is linked with cognitive impairment and hippocampal atrophy [40,41,42]. Indeed, a loss of hippocampal volume in patients with unipolar depression was reported [43], which can be a direct consequence of repeated periods of depression. The hippocampus in depressed patients shows no massive cell loss or pathological changes as in patients with AD [37]. However, a reduced volume in the hippocampus does not mean cell loss as it could be changed in the water content, as was proposed [44,45]. Additionally, the activation of the HPA axis and the cognition problems in AD may be explained by the ongoing process of this disease in the hippocampus, without cortisol being a primary factor. Other studies point out that, from animal models, the pathology of AD is enhanced by exposure to both stress and high levels of cortisol. Indeed, dexamethasone’s treatment increased levels of APP and also promoted the formation of cerebral amyloid plaques [46]. In addition, in wild-type mice, treatment with glucocorticoids, as well as exposure to chronic stress, induced hyperphosphorylation of tau, the initial step in the origin of neurofibrillary tangles [47]. Chronic stress can also induce disruption of the blood–brain barrier (BBB) and increase neuroinflammation that aggravates AD outcomes. This BBB dysfunction causes decreased Aβ entry from the brain to blood circulation, leading to its accumulation [48]. Another study, in mice, demonstrated a relationship between stress exposure and Aβ peptide levels in the brain. Indeed, chronic stress increased the levels of Aβ peptide by 84% [49]. Moreover, in a study with the animal model of AD APPV717I-CT100 (transgenic mice), it was reported that persistent stress has a correlation with pronounced behavioral problems, amyloid deposits extracellularly, and neurodegeneration [50]. Another study revealed that after treatment of triple transgenic AβPP/PS1/MAPT mice with dexamethasone, increased levels of AD biomarkers such as Aβ precursor protein (AβPP) and Aβ were observed [46].

In the past, it was thought that only the dysfunction of the cholinergic system was responsible for the symptoms observed in AD [51]. However, the important role of the monoaminergic system in AD has been increasingly considered by the scientific community, supported by several publications. In particular, the serotonergic system appears to play an important role in both learning and memory retention through interactions with other neurotransmitter systems, such as the cholinergic, GABAergic, dopaminergic, and glutaminergic systems [52,53]. With particular emphasis, recently developed serotonergic antidepressants such as vortioxetine, in addition to inhibiting serotonin transport, are serotonergic receptor antagonists important for cognitive function enhancement, such as in the case of the serotonin 7 receptor (5-HT7). Thus, in patients with depression and mild AD, this drug showed significant improvements in cognitive function compared to the traditional SSRIs, thus becoming a very promising topic of studies [54]. Overall, there is an important influence of the serotonergic system on AD. As stated earlier, stress affects several aspects of serotonergic signaling in the brain and serotonergic drugs can modulate the effects of stress. This leads to the importance of investigating this issue: the study of how serotonin influences the stress response in AD.

### 2.3. In Parkinson’s Disease

The idea that stress is associated with PD has been considered for more than 100 years. It started when Gowers linked anxiety and stress as common antecedents of PD. Additionally, the extreme stress experienced in the Holocaust or during war has been associated with this disease. Stress interferes with the development of PD and can dramatically exacerbate common symptoms, such as rigidity and tremors. There is, indeed, evidence that demonstrates this role [55,56]. In animals, motor performance was worsened and there was a loss of nigral neurons when elevated levels of glucocorticoids and corticosterone were verified [4,57]. In PD patients, cortisol is elevated when compared to healthy controls. There is a connection between HPA axis hyperfunction and dopamine, highlighted by the fact that treatment with levodopa can reduce cortisol levels in PD patients. Indeed, glucocorticoids accelerate disease progression. Additionally, emotional stress can exacerbate the motor symptoms of PD. After exposure to stress, individuals with PD have hedonic responses, to a lesser extent. Indeed, there is a significant relationship between stress, dopaminergic neurodegeneration, and dopamine metabolism and production, namely depletion of dopamine in the nigrostriatal and non-nigrostriatal systems and exacerbated expression of nigrostriatal and non-nigrostriatal α-synuclein [3,54,56]. In another study, after MPTP (1-methyl-4-phenyl-1,2,3,6-tetrahydropyridine) induced neuronal damage and impairment in the acquisition of motor skills in mice, results revealed death of dopaminergic neurons in substantia nigra, as well as exacerbation of PD symptoms, namely motor and learning deficits [58]. Considering all this, it is important to consider these studies and the role of stress in PD to improve future research studies, as well as the clinical care of patients [57].

Symptoms such as depression and anxiety are frequently observed in PD patients. Recently, a study aimed to evaluate the stress-induced effects after treatment with Fluvoxamine (SSRI) on dopaminergic neurons in a mouse model with PD. The study concluded that animals that were exposed to stress had high plasma levels of corticosterone and malondialdehyde, an effect that was attenuated with treatment with Fluvoxamine. Also, this drug appeared to attenuate the vulnerability of dopaminergic neurons to stress and neurotoxic treatments, such as 6-hydroxydopamine (6-OHDA) [59]. Thus, this study suggests the connection between serotonin and the stress associated with PD. In fact, through several studies where animals are stressed by maternal separation, it has been shown that stress affects not only the HPA system but also the serotonergic system present in the hippocampus. Furthermore, the stress causes dysfunction of the central dopaminergic system, being very important in the symptoms of PD, such as motor symptoms [60]. Indeed, there is growing recognition of the serotonergic system in PD. In a study with aged A53T mutation α-synuclein-expressing mice, degeneration of the axons of serotonergic fibers in the prefrontal cortex of these mice, as well as an altered fiber network, were observed. Additionally, increased levels of tryptophan hydroxylase 2 mRNA and 5-HT1B expression were observed in a transgenic animal model for PD, and can be associated with recompense processes within the serotonergic system [61]. Still highlighting the role of serotonin in PD, Pimavanserin is an inverse antagonist of the serotonin 5-HT2A receptor that presents benefits in the context of levodopa-induced psychosis, frequently observed in PD. Having affinity for this receptor and less affinity for D2 or histamine receptors, this drug has the benefit of reducing sedation and motor side effects, being an alternative for patients with PD associated psychosis [62]. Another interesting cross-sectional study about the interplay of serotonin and PD reported that dysfunctions in the serotonergic system may precede the dopaminergic dysfunctions and motor symptoms observed in PD patients that have A53T mutation in α-synuclein. These findings support the use of molecular imaging of serotonergic components (such as transporters) as complements of PD diagnosis [63]. In another study in mice, the transfection of mesenchymal stem cells with an intracellular domain of the *Notch1* gene (important for regulation) demonstrated decreased degeneration of not only dopaminergic, but also serotonergic neurons in patients with PD, providing better outcomes and being a potential cellular therapy [64].

## 3. Immune System Involvement

### 3.1. In Major Depressive Disorder

Some immune cells, such as circulating granulocytes and monocytes, chemokines, and cytokines, can influence the brain directly, influencing neuronal networks that are involved in depression. Indeed, inflammation plays an important role in MDD. Thus, there is a favorable relationship between antidepressant treatments and psychotherapy, and the reduction of inflammatory signs. There is evidence that both the release of cytokines with a proinflammatory character and the stimulation of anti-inflammatory cytokines are inhibited and stimulated by antidepressants, respectively [65,66,67]. Proinflammatory cytokines can activate the HPA axis, resulting in elevated levels of cortisol and glucocorticoid receptor resistance, mechanisms involved in MDD [68]. Also, these proinflammatory cytokines worsen the pathogenesis of MDD by modulating the tryptophan–kynurenine pathway and the synthesis of the quinolinic acid (NMDA receptor agonist) and 3-hydroxykynurenine [69]. Depressed patients with difficulties in response to antidepressant treatment have TfR, IL-6, sIL-6, CRP, sIL-2R, and AGP concentrations elevated in their plasma and may possess abnormal alleles of *IL-1* and *TNF* genes, as well as problems in the normal functioning of T cells [65,67,70,71]. Additionally, meta-analysis studies comparing MDD patients and healthy individuals revealed that in MDD individuals a raise in the levels of molecules such as TNF, IL10, sIL-2 IL-18, IL-12 and IL-1RA was observed, contrasting with a reduction in the levels of interferon-g (IFN-g), versus the healthy controls [72]. At a genomic level, upregulation of the expression of genes that code important mediators in inflammatory pathways (such as *IL-1b, IL-6*) has also been observed in peripheral blood mononuclear cells of patients with MDD [73]. Many studies support the role of microglia activation in psychiatric disorders, such as MDD [74]. An example is a study that demonstrated the presence of increased levels of proinflammatory cytokines followed by microglia activation and consequent transport of monocytes to the brain, leading to anxiety behaviors [74,75]. Moreover, in patients with MDD, the presence of proinflammatory cytokines originated by the microglia (such as TNF-α) reduced the presence of the neurotransmitters serotonin, dopamine, and noradrenaline in several ways, including reducing their synthesis [76]. Moreover, neuronal apoptosis and less levels of neurotransmitters synthesis can result from a chronic activation of microglia, contributing to depressive episodes [77]. Supporting this role of microglia in MDD, it has been proposed that some antidepressants (such as imipramine) have anti-inflammatory effects by reducing microglial activation, thus decreasing the levels of proinflammatory cytokines [78]. In another study, it was also demonstrated that the pathway of glucocorticoid receptor-NF-κB-NLRP3, when activated in microglia, is important to mediate the neuroinflammation induced by chronic stress and depressive behavior. Indeed, the dexamethasone (used to mimic glucocorticoid inflammation) increased NF-κB and NLRP3 levels. Then, after inhibition of NF-κB and knockout of NLRP3, the levels of inflammation and depressive-like behaviors decreased [79]. Another important study revealed that increased levels of TSPO (translocator protein—present in the activated microglia), are present in the anterior cingulate cortex of patients during moderate and severe depressive episodes, highlighting the importance of anti-inflammatory therapies for MDD [80]. The upregulation of *ionized calcium-binding adapter molecule 1* (IBA1) and *monocyte chemoattractant protein 1* (MCP-1) genes and, consequently, increased density of IBA1-positive microglia, was also observed in postmortem brain tissues of highly depressed patients [81].

Several inflammatory diseases, such as rheumatoid arthritis and multiple sclerosis, are characterized by increased risk for depression. For example, patients that have diabetes have twice the risk of developing depression, as well as up to 70% of patients with rheumatoid arthritis or systemic lupus erythematosus [82]. Also, in mice with depressive behaviors, an elevated number of monocytes in the blood has been observed, and its neutralization is enough to get a reduction in depressive behaviors [83]. Additional studies point out that the treatment of mice with lipopolysaccharide (LPS), which induces an innate immune response, triggered an inability to feel pleasure and weight loss. Highlighting the importance of the immune system in depression, mice deficient in the inflammatory caspase-1 also exhibit resistance to LPS-induced depressive behavior [84]. Another study revealed that the expression of HMGB1-RAGE (high mobility box 1 protein—receptor for advanced glycation end products) in microglia persistently increased the likelihood of developing depressive episodes, mainly after chronic levels of stress [85]. At the microbiome level, there are also differences between healthy and depressed individuals, contributing to dysregulated immune responses [86]. For example, *Coprococcus* and *Dialister* bacteria are much less present in MDD patients versus healthy controls. These bacteria are important for the normal regulation of innumerous functions. *Coprococcus* interacts with dopamine pathways, normally affected in depressed individuals. Nevertheless, this bacterium also produces butyrate, an anti-inflammatory molecule [87]. Studies that focused on the relationship of non-steroidal anti-inflammatory drugs (NSAIDs) as candidates to the antidepressive therapy revealed that, overall, this class of drugs produces a pronounced reduction in depressive behaviors, with a special spotlight on inhibitors of cyclooxygenase-2 (COX-2), such as celecoxib [88]. In another study, increased levels of anhedonia were also associated with decreases in the connection between the prefrontal cortex and striatum. These findings were also associated with increased levels of proinflammatory cytokines and C-reactive protein in MDD patients [89]. An intense exploration of the connection between the immune system and MDD will, certainly, enrich our understanding of this prevalent and complex disease, designing better treatments and improving the life quality of patients [90]. Keeping in mind that innate and adaptive immunity are dysregulated in patients with MDD, a good therapeutic strategy involves controlling these inflammatory processes [91].

In addition to all its known roles, serotonin also has important functions in the immune system. Several studies have shown that, in fact, different types of immune cells, such as T cells, produce, store, and respond to serotonin. This connection has also been linked to mood disorders [92]. Studies that evaluated the role of SSRI treatment in autoimmune diseases and the consequent influence on the Th17:Treg ratio, demonstrated that the modulation of serotonin levels with SSRI treatment decreased this ratio and, therefore, these drugs seem to have an anti-inflammatory action [92,93,94]. In the context of MDD, it has been shown that patients with this pathology have high levels of this Th17:Treg ratio, having a low percentage of Treg compared to patients without this pathology [95,96,97]. In this context, treatment with SSRIs, in general, led to an increase in these types of T cells, thus leading to a normalization of the Th17:Treg ratio to levels identical to healthy controls [95]. Other studies have also demonstrated this relationship, namely a study in which mice were treated with rapamycin, a drug whose function is to increase Treg cells. In this study, the animals treated with this drug showed enhanced cognitive functions and increased levels of serotonin and dopamine compared to controls [92]. However, despite this evidence, further studies on this topic are still needed. For example, different SSRIs can modulate the immune response differently from each other and, therefore, further research on the relationship of serotonin with the immune system is crucial [92,98].

### 3.2. In Alzheimer’s Disease

Several immune system disorders are considered risk factors for AD. Mainly because of the inflammatory component of the illness, with a special highlight in IgA, the immune system is dysregulated in patients diagnosed with AD. Individuals with or without AD have shown different levels of IgA. In a particular study, the levels of IgA in AD patients and patients without AD were 103.97 ± 65.62 and 23.79 ± 16.1, respectively. These data indicate that AD patients suffer from an immune alteration [99]. Indeed, neuroinflammation is considered a critical characteristic of AD. Neuritic plaques composed of Aβ and neurofibrillary tangles are, indeed, surrounded by astrocytes and microglia with reactive characteristics [100]. This microglia is known to release proinflammatory factors. Examples include the tumor necrosis factor-alpha (TNFα) and interleukin 1 beta (IL-1β) [101,102], highlighting the role of neuroinflammation in AD. Additionally, the interplay between the immune system and AD was demonstrated by the attachment of complement proteins to the damaged tissue and by the activation of cells that are associated with the immune system [103,104]. In fact, a research study focused on complement C3, an immune system molecule which helps microglia in the clearing of the plaques and is up-regulated in AD, contributing to the synapse loss that leads to cognitive decline. It was demonstrated that knocking out the gene of this molecule in mice models of AD, improved the animals’ performance in both learning and memory tests, despite them having more plaques in their brains and fewer and less activated microglia [105]. Another study aimed to analyze amyloid-beta stimulated T lymphocytes in AD patients versus mild cognitive impairment in healthy individuals. The results demonstrated that Aβ stimulated CD4(+) T cells that produce IL-21 and IL-9, and that express the RORγ and NFATc1 transcriptional factors, as well as monocytes that produce IL-23- and IL-6-, were significantly increased. On the other hand, IL-10-producing monocytes were in a low number in AD, compared with the other conditions [106]. Other studies also point out that various proinflammatory cytokines such as TNFα, IL-1β, and IL-18 and anti-inflammatory cytokines, like interleukin-1 receptor antagonists were increased in cerebrospinal fluid of patients with AD, demonstrating an immune disturbance in patients with this disease [107,108,109]. Genomic studies have also associated AD with dysregulation in the innate immune system and uncontrolled inflammatory processes. However, the exact mechanisms by which innate immunity influences AD remain elusive [110]. Th17 cells are also very important for the pathogenesis of AD and their involvement in neuroinflammation observed in AD patients has been studied. For example, this involvement was observed in AD rodent models, inducing neurodegeneration of aβ1–42. Through the disrupted BBB, these cells can infiltrate into the brain, resulting in the production of proinflammatory cytokines such as Il-22 and IL-17 [111]. Indeed, in another study in mice models of AD, the effects of IL-17 neutralizing antibody (IL-17Ab) reduced neurodegeneration, improved memory, and decreased proinflammatory factors, highlighting the importance of Th17 cells in AD [112]. Another study linked the impairment of microglial TREM2 (Triggering Receptor Expressed On Myeloid Cells 2) signaling with reduced levels of neuroinflammation and neurodegeneration, important in the context of AD [113]. Thus, a better understanding of these processes may facilitate the study of novel therapeutic strategies.

As already mentioned, neuroinflammation is a marked feature in AD. Studies have shown that treatment with SSRIs reduced the number of cytokines in the circulation, as well as attenuated several inflammatory pathways typically elevated in this pathology. Thus, a connection between serotonin, neuroinflammation, and AD is evident [114,115]. Studies have shown that changes in the function of the serotonin transporter are caused by proinflammatory cytokines elevated in AD, such as interleukin-1 beta [116]. Also, in studies with neuronal cell lines and in vivo studies, TNF has been shown to increase the maximum uptake rate of serotonin [117]. In turn, treatment with IL-6 reduced the levels of SERT mRNA in the rat hippocampus. Thus, these data demonstrate that, during AD, these cytokines interfere with the serotonergic system in specific ways for each cytokine, with the need for studies in this area [118]. However, it is important to keep in mind that the research about this topic highlights that the progression of cerebellar amyloidosis, a characteristic of AD, is associated with neuroinflammation, which is mirrored in events such as changes in integrity and pre-synaptic serotonergic activity [116]. Another study linked AD, depression, and the immune system. Indeed, this study reported that accumulation of Aβ oligomers and toxins present in AD patients lead to depressive episodes in mice through microglial activation, alterations in the TNF-α signaling pathway, and reduced presence of serotonin in the brain. In this study, the authors revealed that serotonin decreases the activation of microglia, a negative regulator of these cells. Additionally, this study demonstrated that in Toll-like receptor 4-deficient mice, the presence of Aβ oligomers did not induce depressive episodes. Supporting the relationship between AD and serotonin, it was observed that disturbances in serotonin signaling pathways induced by Aβ peptide promote brain inflammation. On the other hand, serotonin can prevent the activation of microglial cells that are induced by Aβ [119]. Additionally, the SSRI administration to animal models of AD, increased levels of serotonin resulted in lower Aβ production, supporting the idea that serotonin induced pathways influence Aβ deposits in a negative way [120].

### 3.3. In Parkinson’s Disease

A significant amount of evidence highlights a role for both the innate and adaptive immune systems in the pathophysiology of PD [121]. Indeed, postmortem analysis of brains from PD patients shows adaptive immunity and microglia activation as contributors to the disease progression [122,123]. Now, it is known that, in response to α-syn aggregation and toxicity, microglia activation occurs in the initial stages of the disease, being critical in clearing aggregates of α-syn and, thus, initiating a series of inflammatory responses. This activation of microglia by α-syn also leads to infiltration of monocytes and macrophages through the CCL2- CCR2 process. Also, respectively, an adaptive immune response through CD8 or CD4 will be initiated by the fact that peptides of α-syn will be presented by neurons through MHCI and by microglia, monocytes, or macrophages through MHCII. In response to the activation of CD4-Th cells, cytokines are produced which may lead to a potentiation of proinflammatory events, mainly if a differentiation into Th1 or Th17 cells occurs [124]. In fact, studies found that CD8+ and CD4+ T cells were present at higher levels in PD patients [125]. In another study, in human blood samples of PD patients, a reduced number of lymphocytes (overall) was observed, whereas CD8 + T cells increased, as well as IFN-γ/IL-4 ratio, comparing to healthy controls [126]. Additionally, in a research study, the concentrations of IL-1 beta and IL-6 in the dopaminergic, striatal regions were significantly higher in patients with PD in comparison to controls [127]. On the other hand, the production of reactive oxygen and nitrogen species and cytokines is likely to promote apoptotic signals that compromise the survival of neurons. Additionally, the production of chemokines by microglia has a critical role in triggering the infiltration of immune cells into the central nervous system, which may be important in disease progression and affect neuronal health [128,129]. Many studies are highlighting the important role of astrocytes in PD neuroinflammation. These cells become reactive and proinflammatory in response to several signals, such as TNF—α and C1q secreted by the microglia [130]. In agreement with this, in postmortem tissues of PD patients, the proinflammatory phenotype of the astrocytes has been observed [131]. Hereditary cases of PD are linked to mutations in DJ-1 in 1% of the cases. This molecule has been studied, and studies report that the knockdown of this molecule in microglia leads to an increase in microglia’s neurotoxicity, mainly due to dopaminergic neurons. Additionally, in DJ-1 knockdown microglia, an increase in the production of IL-6 and IL-1β cytokines, stimulated by α-Syn, was reported. In these microglia, impairment in autophagic processes, affecting α-Syn clearance, was also observed [132].

Neuroinflammation is a characteristic that defines PD. In the context of neuroimmunology, dopamine, in spite of being a neurotransmitter, also plays an important role in the regulation of cells in the immune system. The dopamine transporter, known as DAT, exists in lymphocytes and other cells of the immune system, such as monocytes and macrophages, thus demonstrating a relationship between the immune system and the dopaminergic system that is extremely relevant to the pathophysiology of PD [133]. Besides this relationship between dopamine and neuroinflammation present in PD, a relationship between serotonin and neuroinflammation associated with this disease is also considered, despite scarce studies in this field. However, knowing that there is a relationship between serotonin and the immune system, as discussed throughout this article, and knowing that serotonin plays important roles in PD, such as the fact that cortical Aβ peptide amount in patients with PD associates in an inverse way with serotoninergic innervation [134], it is deduced that a relationship between these three factors is an important focus for future research. A deeper understanding of the immune response and how it interplays with PD will certainly lead to the development of novel and more effective immunotherapies, improving the life quality of patients.

## 4. An Interplay between Stress and Immune System

**Figure 3:**
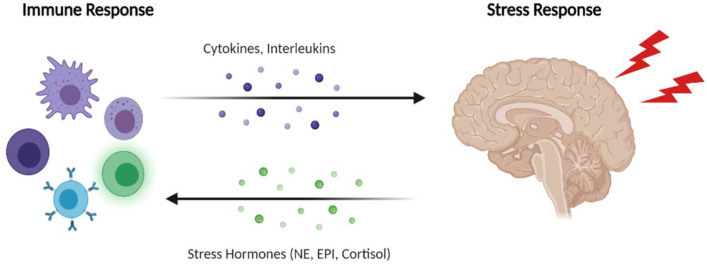
Connection between immune and stress responses. The immune system and the stress response are linked through various players, such as interleukins, cytokines, and various hormones. This link between these systems can be important in the pathophysiology of several diseases, such as AD. Created with BioRender.com.

Many systems in the body are affected by stress, the immune system being no exception (Figure 3). This system has key roles during the stress response, enhanced or suppressed by a variety of stressors. In fact, stress triggers a wide range of inflammatory activities important in a variety of pathologies. An example is the case of the immune response induced by psychological stress and risk of AD in individuals that have experienced traumatic conditions, such as war or loss [2,135,136]. Particularly, an elevation in IL-1 and IL-6 concentrations are related to the stress response, important to enhancing the immune system and contributing to survival. Indeed, brain functions are modulated by cytokines, where they can regulate processes like the stress response, as a bidirectional response. Acute stress leads to a redistribution of immune cells in the body, enhancing functions like immune surveillance. Additionally, norepinephrine (NE), cortisol, and epinephrine (EPI) (stress-related hormones) influence the immune system. As an example, norepinephrine leads to an increase in leucocyte numbers and the mobilization of immune cells to enter the blood. However, chronic stressors are associated with the suppression of both cellular and humoral immunity [137]. Indeed, microglial activation after stress conditions leads to the release of norepinephrine, which activates several signaling pathways in many immune cells, including microglia itself. This evidence is supported by the fact that β-adrenergic receptor antagonists can impair this mechanism of microglia activation [138]. By inducing cytokine production, stress also induces the (IDO—indoleamine 2,3-dioxygenase)/kynurenine pathway, thus interplaying with the immune system. IDO is important in the process of the catabolism of tryptophan, leading to reduced levels of serotonin which is produced from tryptophan. These reduced levels of serotonin thus promote depressed states. By the action of a huge range of proinflammatory cytokines (such as TNF and IL-1b), IDO is activated in cells like glial cells and macrophages [139]. In patients with MDD, problems in the functioning of the BBB were already described [140]. After stress conditions, the opening of the BBB may lead to the infiltration of immune cells. It has been demonstrated that after stress, T cells and monocytes infiltrate the brain. In depressed mice, Th17 cells can accumulate in several brain areas, such as the hippocampus, promoting depressive behaviors [141,142]. Another study that supports a relationship between stress and the immune response is a study where authors investigated the effect of the knockout of the *Cx3cr1* gene in microglia, a gene that is important in the regulation of microglia. The results obtained demonstrated that this gene is important in the stress response coordinated by the microglia and its knockdown prevents the effects of stress and depressive behaviors in mice [143]. Additionally, in Cx3cr1-GFP reporter mice, microglia phagocytosed more synaptic and neuronal material after exposure to chronic levels of stress. Another study revealed that, after exposing mice to different types of chronic stress, many alterations occurred, namely the loss of hippocampal endogenous microglia and reduction of process lengths and activations markers of these cells. This process of hippocampal microglia loss was previously described as important to mediate the development of MDD in mice [144]. Moreover, another study in rats revealed that a pronounced increase in the density of Iba1+ microglial cells was observed in pre-puberty offspring after prolonged maternal sleep deprivation. These findings were accompanied by deficits in neurogenesis and memory impairment. After the treatment with minocycline, these alterations were reversed. This is explained by the fact that this drug prevents microglial transformation, highlighting a relationship between stressful events (such as sleep deprivation) and alterations in the immune system [145]. After LPS (lipopolysaccharide) administration in pregnant mice, a study demonstrated that prenatal stress induced modifications in microglia of offspring, leading to vulnerability to depressive behaviors. Indeed, stressed animals revealed increased proportions of Iba1-immunoreactive cells, mainly in the hippocampus [146]. After exposure to high levels of stress, in wild-type mice, a loss of dopaminergic neurons and reduced levels of IBA1-positive microglial present in the substantia nigra was also reported [147].

Another important mediator in the response to stress is the ATP/P2X7R-NLRP3 inflammasome pathway, sensed by the innate immune system. Recently, it was reported that administration of antagonists of P2X7 receptors blocked the release of stress-induced cytokines (such as TBF-α and IL-1β) through this cascade, reducing the inflammatory response and being an interesting treatment approach for stress-related disorders such as MDD [148]. Furthermore, in the gene coding this receptor, a single nucleotide polymorphism (Gln460Arg) was associated with an increased risk of depression [149]. In another study, when depressive behaviors in mice were reversed, colony-stimulating factor 1 reversed dystrophy in microglia in the hippocampal area, demonstrating the role between microglial functional changes and depression-like behaviors [150].

The connection between stress and immune functions makes this an interesting subject of research, important for future studies to gain a better understanding and, thus, new advances in medicine [151].

## 5. Conclusions

Neurological diseases, such as the aforementioned AD, PD, and MDD, are being increasingly studied. This intense investigation into these prevalent diseases has generated more knowledge and, above all, the possibility of better outcomes and treatments regarding these conditions. Immune conditions and stress, as well as their interplay and connection with neurotransmitters such as serotonin, are factors with substantial importance in these diseases. Therefore, they can be important targets for the design of new drugs or improvements in already existing drugs. For this, much study has yet to be undertaken in this area, which has gained huge relevance today. These studies will undoubtedly help to better understand these diseases and, above all, improve the quality of life of patients.

## Data Availability

Not applicable.
